# Harmine, a dual-specificity tyrosine phosphorylation-regulated kinase (DYRK) inhibitor induces caspase-mediated apoptosis in neuroblastoma

**DOI:** 10.1186/s12935-018-0574-3

**Published:** 2018-06-07

**Authors:** Katie L. Uhl, Chad R. Schultz, Dirk Geerts, André S. Bachmann

**Affiliations:** 1 Department of Pediatrics and Human Development, College of Human Medicine, Michigan State University, 400 Monroe Avenue NW, Grand Rapids, MI 49503 USA; 2 Department of Medical Biology, Academic Medical Center, University of Amsterdam, 1105 AZ Amsterdam, The Netherlands

**Keywords:** Apoptosis, DYRK2, Harmine, Molecular docking, Natural products, Neuroblastoma

## Abstract

**Background:**

Neuroblastoma (NB) is an early childhood malignancy that arises from the developing sympathetic nervous system. Harmine is a tricyclic β-carboline alkaloid isolated from the harmal plant that exhibits both cytostatic and cytotoxic effects. Harmine is capable of blocking the activities of dual-specificity tyrosine phosphorylation-regulated kinase (DYRK) family proteins and mitogen-activated protein kinase. These kinases promote proliferation and inhibit apoptosis.

**Methods:**

Four human NB cell lines were used to study the effects of harmine treatment: SKNBE and KELLY (*MYCN*-amplified) as well as SKNAS and SKNFI (*MYCN* non-amplified). The anti-cancer properties of harmine were examined by RealTime-Glo MT cell viability assays, caspase activity assays, PARP cleavage using Western blot analysis, and flow cytometry-based Annexin V detection. A molecular interaction model of harmine bound to the DYRK2 family kinase was generated by computational docking using X-ray structures. NB tumors from human patients were profiled for DYRK mRNA expression patterns and clinical correlations using the R2 platform.

**Results:**

The IC_50_ values for harmine after 72 h treatment were 169.6, 170.8, and 791.7 μM for SKNBE, KELLY, and SKNFI, respectively. Exposure of these NB cell lines to 100 μM of harmine resulted in caspase-3/7 and caspase-9 activation as well as caspase-mediated PARP cleavage and Annexin V-positive stained cells, as early as 24 h after treatment, clearly suggesting apoptosis induction, especially in *MYCN*-amplified cell lines. Elevated *DYRK2* mRNA levels correlated with poor prognosis in a large cohort of NB tumors.

**Conclusion:**

Harmine is a known inhibitor of DYRK family kinases. It can induce apoptosis in NB cell lines, which led us to investigate the clinical correlations of *DYRK* family gene expression in NB tumors. The patient results support our hypothesis that DYRK inhibition by harmine and the subsequent triggering of caspase-mediated apoptosis might present a novel approach to NB therapy.

**Electronic supplementary material:**

The online version of this article (10.1186/s12935-018-0574-3) contains supplementary material, which is available to authorized users.

## Background

Neuroblastoma (NB) is an early childhood malignancy that arises from the developing sympathetic nervous system, resulting in aggressive tumor formation in the sympathetic ganglia and/or the adrenal glands [1]. Amplification of the *MYCN* gene, leading to over-expression of the MYCN protein, is the most prevalent NB genetic aberration. It is found in ~ 20% of NB, predominantly in high-stage tumors and has been linked to high risk disease, and poor patient prognosis [1, 2]. Of those high risk patients that respond initially to chemotherapy, the majority will succumb to the disease after a relapse into a chemotherapy-resistant state [2]. In addition to *MYCN* gene amplifications, mutations in genes encoding the mitogen-activated protein kinase (MAPK) pathway, and in the ALK gene have been identified as main drivers in the majority of NB [1].

The harmal plant (*Peganum harmala* L., family Zygophyllaceae), also called Syrian rue, is a perennial shrub native to the eastern Mediterranean region. Various parts of the plant have long been used in traditional folk medicine [3]. Harmine is a tricyclic β-carboline alkaloid isolated from harmal seeds and acts as a monoamine oxidase A (MAO-A) inhibitor [3–5]. Intake of alkaloids from the harmal plant can have anti-depressive, analgesic, and anti-bacterial pharmacological effects [6–8].

Harmine has been shown to induce apoptosis and inhibit cell proliferation, migration, and invasion in a dose-dependent manner in various human cancer cell lines including C334, CCD18LU, HeLa, HL-60, K562, SW480, BGC-823, and SGC-790 [9]. While lower concentrations of harmine typically induced cytostasis, higher concentrations are associated with cytotoxicity [10].

The dual-specificity tyrosine phosphorylation-regulated kinase (DYRK) family proteins are related to the MAPK family. However, the activating tyrosine phosphorylation of DYRK family kinases is not catalyzed by upstream kinases but occurs through autophosphorylation [10]. There is growing interest in the role of DYRK family kinases in cancer, as they can act as regulators of protein stability during the cell cycle and regulate the activity of the proteasome [11, 12]. Remarkably, harmine inhibits all DYRK family members (DYRK1A, DYRK1B, DYRK2, and DYRK4), with the highest affinity for DYRK1A [13, 14].

In this study, we identified that harmine induces apoptotic cell death in NB cells, generated a molecular interaction model for harmine bound to DYRK2, and showed that *DYRK2* mRNA expression patterns in a large cohort of human NB tumors suggest the involvement of DYRK2 in NB tumorigenesis. Together, our results offer a potential new route of NB therapy.

## Methods

### Chemicals

**Fig. 1:**
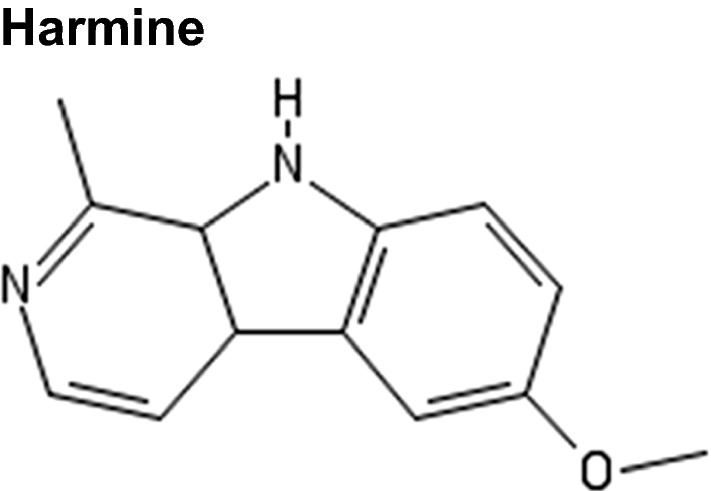
Chemical structure of harmine. Harmine is a β-carboline alkaloid present in *Peganum harmala* plant, and several other Mediterranean plant species. Harmine has a number of pharmaceutical characteristics including irreversible inhibition of monoamine oxidase A (MAO-A), and demonstrates cytotoxicity in various cancer cell lines. The molecular weight of harmine is 212.25 g/mol

Harmine (Fig. 1), LDN-192960, and INDY were purchased from Cayman Chemical. The compound solids were stored at − 20 °C. Stock solutions were prepared by dissolving harmine (100 mM), LDN-192960 (40 mM) and INDY (40 mM) into sterile DMSO (VWR). Stock concentrations were filtered prior to being added to the cell cultures.

### Cell culture

The human NB cell lines KELLY (also known as N206; #92110411 from Sigma), SK-N-AS (called SKNAS in this study; CRL-213 from ATCC), SK-N-BE (clone SKNBE(2)C, called SKNBE in this study; CRL-2268 from ATCC), and SK-N-FI (called SKNFI in this study; from the Children’s Oncology Group) were cultured in RPMI 1640 (VWR), supplemented with 10% heat-inactivated fetal bovine serum (Invitrogen), Penicillin (100 IU/mL) and Streptomycin (100 μg/mL) (30-002-CI, Corning). All cells were purchased from their respective suppliers within the last 2 years. The cells were maintained at 37 °C in a humidified atmosphere containing 5% CO_2_.

### Cell viability assay

Cell viability and IC_50_ was determined using the RealTime-Glo MT cell viability assay (G9712, Promega). This reagent allows continuous measurement of cell viability in the same well. Cells were plated at a density of 4000 cells/well into white-walled, opaque assay plates. After the plated cells had been given 24 h to adhere, they were treated with harmine concentrations ranging from 0 to 1 mM. The MT Cell Viability Substrate and NanoLuc Enzyme were equilibrated to 37 °C, 2× RealTime-Glo reagent was prepared, and an equal volume was added to each well. For time zero measurements, cells were incubated with reagent for 20 min at 37 °C, and luminescence was measured on a Biotek Synergy microplate reader. Luminescence was measured at 24, 48, and 72 h after addition of harmine.

### Cytotoxicity assay

The colorimetric SRB assay was used to measure cytotoxicity as previously described, following the treatment with DYRK2 inhibitor LDN-192960 or DYRK1A/B inhibitor INDY [15–17]. Briefly, NB cells were plated in transparent flat 96-well plates and allowed to attach overnight. At the initiation of each experiment (t = 0) and after drug treatments, cells were fixed with 10% TCA at 4 °C for 1 h, washed with deionized water, and dried at room temperature. Cells were then stained with 100 μl of 0.4% SRB in 1% acetic acid for 20 min at room temperature, rinsed five times with 1% acetic acid and allowed to dry at room temperature. One hundred µl of 10 mM Tris–HCl pH 7.0 was added to each well, shaken for 10 min at room temperature and read at 540 nm using a Molecular Devices Flexstation 3 microplate reader.

### Caspase activity assays

The quantification of the relative caspase activity in harmine-treated cells was carried out using the Caspase-Glo 3/7 and Caspase-Glo 9 Assay kits (G8091 and G8211, respectively, from Promega). Cell lines were plated at a density of 16,000 cells/well into white-walled, opaque 96-well plates. Twenty-four hours after plating, cells were treated with 0, 25, 50 and 100 µM harmine for 24 h. Caspase Glo reagents were added to the cells and incubated at room temperature. Luminescence was measured using a Biotek Synergy microplate reader every 20 min for 3 h.

### Western blot analysis

Whole cell lysates were prepared using radioimmunoprecipitation assay (RIPA) buffer, (20 mM Tris–HCl [pH 7.5], 135 mM NaCl, 2 mM EDTA, 0.1% (w/v) sodium lauryl sulfate, 10% (v/v) glycerol, 0.5% (w/v) sodium deoxycholate, and 1% (v/v) Triton X-100). The RIPA buffer was supplemented with cOmplete™ Protease Inhibitor Cocktail (Roche), and 0.27 mM Na_3_VO_4_ and 20 mM NaF as phosphatase inhibitors. Protein concentration was determined using the Bradford dye reagent protein assay (Bio-Rad). Equal amounts of protein were resolved using 12% SDS-PAGE, and transferred to 0.45 µM polyvinylidene difluoride Immobilon-P membrane (Millipore). The transferred proteins were incubated with the following primary antibodies: PARP rabbit polyclonal antibody (#9542) from Cell Signaling Technologies at 1:1000 dilution and GAPDH mouse monoclonal antibody (#SC-47724) from Santa Cruz Biotechnology at 1:1000 dilution. After incubating the blots with the primary antibody at 4 °C for at least 8 h, they were washed and incubated with secondary antibodies at 1:10,000 dilution for 1 h at room temperature. The secondary antibodies used were LiCor IRDye^®^ 680RD Goat anti-Mouse IgG, (925-68070), and LiCor IRDye^®^ 680RD Goat anti-Rabbit IgG, (925-68710).

### Annexin V detection

To quantify the percentage (%) of apoptosis induction in harmine-treated NB cells, the Annexin V Detection Kit APC (88-8007, Invitrogen) assay was used. Samples were prepared according to the manufacturer’s protocol and analyzed by flow cytometry. In brief, the cells were plated overnight and then treated with 0 μM or 100 μM harmine for 24 h at 37 °C. Cells were collected by centrifugation and stained with Annexin V APC/DAPI staining solution at room temperature for 20 min in the dark. Next, cells were collected by centrifugation suspended in PBS (pH 7.4) and analyzed immediately using flow cytometry Excitation/emission for Annexin APC and DAPI were 633/700 and 350/450 nm respectively.

### Molecular docking of DYRK2 and harmine

The molecular docking model for DYRK2 in complex with harmine was constructed by identifying the target structures from the Protein Data Bank (PDB) [18]. The crystal structure of human DYRK1A (PDB ID: 3ANR) in complex with harmine was previously published [13, 18]. To predict if DYRK2 also forms a complex with harmine, we generated a molecular docking model with coordinates from an existing crystal structure of DYRK2 in complex with Leucettine (PDB:4AZF) using the online docking web server SwissDock (http://www.swissdock.ch/) [18–21]. Chain A of 4AZF was isolated from the original crystal structure, and the Leucettine L41 ligand was removed. The modified structure was then entered into SwissDock, along with the chemical structure of harmine (ZINC ID: 27646846) taken from the ZINC molecule database (http://zinc.docking.org/) [19, 20]. The conformational ΔG was calculated and deemed best fit by the parameters set by SwissDock. The resulting molecular interaction model was visualized using visual molecular dynamics (VMD) [22].

### NCBI BLAST sequence alignment

Alignment of the DYRK family sequences was carried out using the Blastp tool provided by the National Center for Biotechnology Information (NCBI) [23, 24]. The amino acid sequences for human DYRK1A and human DYRK2 used for the alignment were taken from the Basic Local Alignment Search Tool (BLAST) database, (Accession No. Q13627 and AAH06375, respectively) (https://blast.ncbi.nlm.nih.gov/Blast.cgi) [18, 24]. The query cover reported herein was given as part of the output of the Blastp program, as was the percentage of identity and the corresponding E-value.

### NB public mRNA expression dataset analysis

For analysis of *DYRK* gene expression in human NB patients, the largest public NB cohort for which genome-wide tumor RNA-sequencing has been performed, SEQC-498, (n = 498; GSE62564) was analyzed using the R2 genomics analysis and visualization platform developed in the Department of Oncogenomics at the Academic Medical Center—University of Amsterdam (http://r2.amc.nl). Expression data for the datasets were retrieved from the public Gene Expression Omnibus (GEO) dataset on the NCBI website (http://www.ncbi.nlm.nih.gov/geo/) and analyzed as previously described [25].

### Statistical analyses

**Fig. 2:**
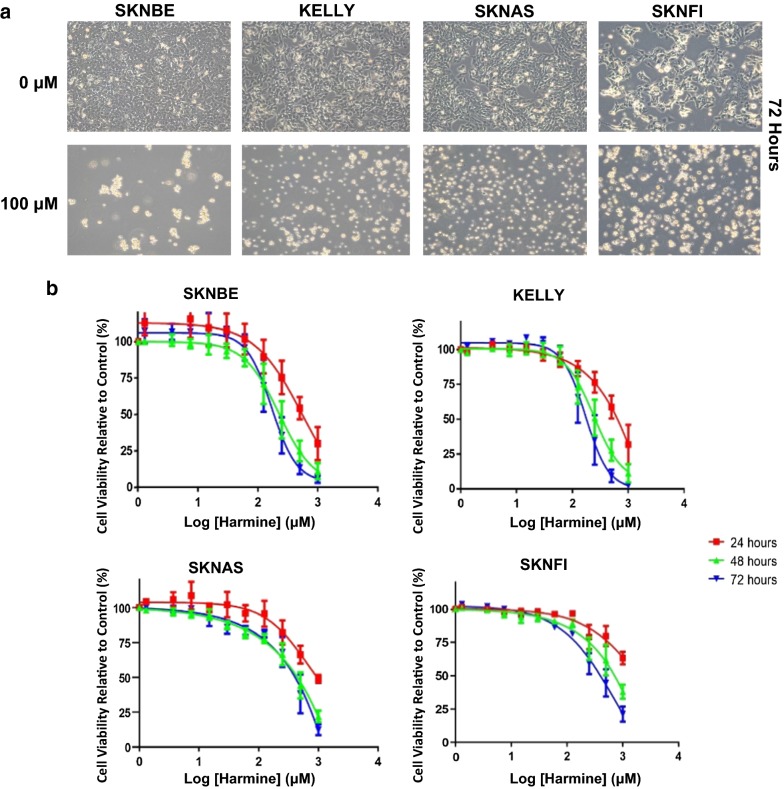
Harmine induces NB cell death. **a** NB cell lines (SKNBE, KELLY, SKNAS, and SKNFI) were treated with 100 μM harmine or left untreated for 24–72 h. Representative micrographs show the effects of 100 μM harmine on cell morphology after 72 h (see Additional file 1: Fig. S1 for micrographs representative of harmine treatments after 24 and 48 h). **b** IC_50_ curves representing cell viability measurements using Real-Time Glo (Promega). NB cell lines were continuously exposed to a range of harmine concentrations (0–1 mM) for a total of 72 h, with measurements after 24, 48, and 72 h. Values were normalized to control and represent the average of three independent experiments ± S.D. (n = 3)

**Fig. 3:**
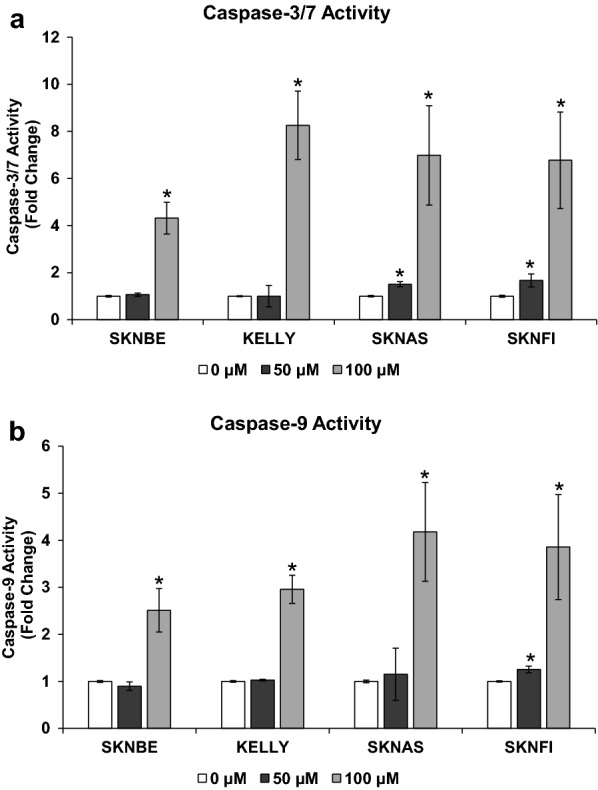
Harmine activates caspase-3/7 and caspase-9 in NB cells. NB cell lines (SKNBE, KELLY, SKNAS, and SKNFI) were treated with harmine (0, 50, 100 μM) for 24 h and caspase activities measured. **a** Caspase-3/7 and **b** caspase-9 are significantly activated in the presence of harmine (100 μM). Results were obtained using the Caspase-Glo Assay Systems as stated in the “Methods” section. The results were normalized to the control value of each cell line; the fold change for each cell line is shown. Data represent the average of three independent experiments, each performed in duplicate ± S.D. (n = 6). The change in activation was calculated using an unpaired student’s t-test, assuming the null hypothesis. Asterisk denotes statistically significant changes in caspase activity compared to control (P < 0.05)

**Fig. 4:**
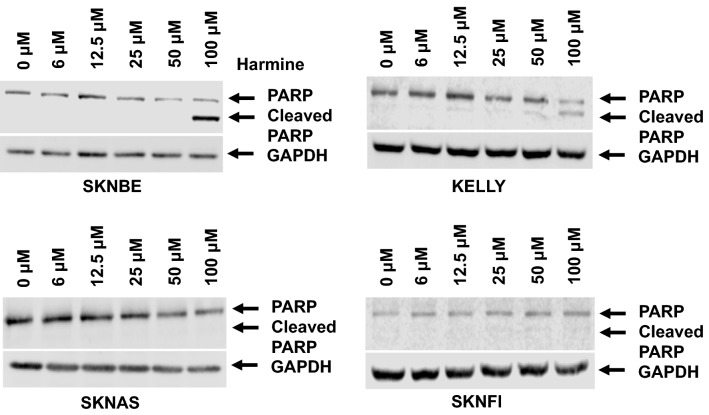
Harmine induces PARP cleavage in NB cells. NB cell lines (SKNBE, KELLY, SKNAS, and SKNFI) were exposed to increasing concentrations of harmine, (0–100 μM) for 24 h and probed for PARP cleavage, an indicator of progressive apoptosis. Whole cell lysates were collected using RIPA lysis buffer and analyzed for PARP cleavage using Western blot. Data are representative of three independent experiments (n = 3)

The statistical significance for the cell viability measurements (Fig. 2b) was calculated using the GraphPad Prism 7 package (https://www.graphpad.com/) and an unpaired student’s t-test assuming the null hypothesis was performed. The change in caspase activity was quantified by calculating the average fold change, after normalizing experimental samples to their respective controls. The significance of both caspase activation and Annexin V apoptotic measurements (Figs. 3, 4) was calculated using an unpaired student’s t-test, assuming the null hypothesis. *DYRK* gene NB tumor mRNA expression correlation with survival probability (Fig. 7) was evaluated by Kaplan–Meier analysis using the log-rank test as described [26]. To determine the optimal value of gene expression to set as cutoff value, all tumor samples were first sorted according to gene mRNA expression and subsequently divided into two groups. Analyses were performed on groups separated by median or average tumor mRNA expression values. *DYRK* mRNA expression correlation with tumor *MYCN* gene amplification was determined using the non-parametric, rank-based Kruskal–Wallis test. For all tests, a P-value < 0.05 was considered to be statistically significant.

## Results

### Harmine induces dose- and time-dependent NB cell death

To study the effect of harmine on the morphology of NB cells, four NB cell lines were exposed to 100 μM harmine. As early as 24 h after treatment, the plated cells began to show signs of morphological changes associated with apoptosis (Additional file 1: Fig. S1). In comparison to control cells, treated cells were smaller in size, rounded in shape, and more detached from the plate surface. After 72 h, the majority of treated cells had become spherical in shape and had detached (Fig. 2a). To determine if harmine induces dose- and time-dependent cell death, the four NB cell lines were treated with increasing drug concentrations (0, 6, 12.5, 25, 50, and 100 μM) and cell viability was measured after 24, 48 or 72 h of treatment (Fig. 2b). Increased treatment length with harmine caused decreased IC_50_ in all four cell lines. The IC_50_ values for SKNBE, KELLY, and SKNFI after a 72 h treatment of harmine were 169.6 ± 0.10, 170.8 ± 0.10, and 791.7 ± 0.77 μM, respectively. The IC_50_ value of SKNAS after 72 h could not be calculated (for all IC_50_ values including 24 and 48 h time points; see Additional file 1: Table S1). The IC_50_ decrease was consistent with the morphological changes observed in the treated cells (Fig. 2a). Moreover, the IC_50_ values suggest that harmine is more toxic to *MYCN*-amplified NB cell lines (SKNBE and KELLY), than to NB cell lines with a normal *MYCN* gene copy number (SKNAS and SKNFI).

### Harmine activates caspase-3/7 and caspase-9 in NB cells

The dose- and time-dependent cell death and associated morphological changes in response to harmine treatment prompted us to determine if harmine induces caspases, known to be activated during apoptosis. NB cells were treated with 0, 50, and 100 μM of harmine for 24 h, after which caspase activity was measured using the Promega Caspase-Glo 3/7 and Caspase-Glo 9 Assay kits. Caspase-3/7 activity increased with increasing harmine concentration (Fig. 3a) and this was significant for all four cell lines at the highest concentration (100 μM). The average fold changes for the caspase-3/7 activity were: SKNBE = 4.32 (P < 1.0 × 10^−4^), KELLY = 8.26 (P < 1.0 × 10^−4^), SKNAS = 6.98 (P < 1.0 × 10^−4^), and SKNFI = 6.77 (P < 1.0 × 10^−4^). Under identical cell treatment conditions, a significant increase in caspase-9 activation was observed in all four cell lines (Fig. 3b). The average fold changes for caspase-9 activity were: SKNBE = 2.51 (P < 1.0 × 10^−4^), KELLY = 2.96 (P < 1.0 × 10^−4^), SKNAS = 4.18 (P < 1.0 × 10^−4^), and SKNFI = 3.86 (P < 1.0 × 10^−4^). The results suggest that harmine triggers apoptotic cell death in NB cells.

### Harmine induces progressive apoptosis in NB cells

To confirm that caspase activation leads to progressive apoptosis, harmine-treated NB cells were analyzed for caspase-mediated PARP cleavage. As shown in Fig. 4, cleaved PARP appeared in whole cell lysates of harmine-treated SKNBE and KELLY cells, but not SKNAS or SKNFI cells. These results confirm that harmine induces apoptosis, with a profound effect on *MYCN*-amplified NB cells (SKNBE and KELLY); both cell lines representing the most aggressive sub-types of NB tumors.

**Fig. 5:**
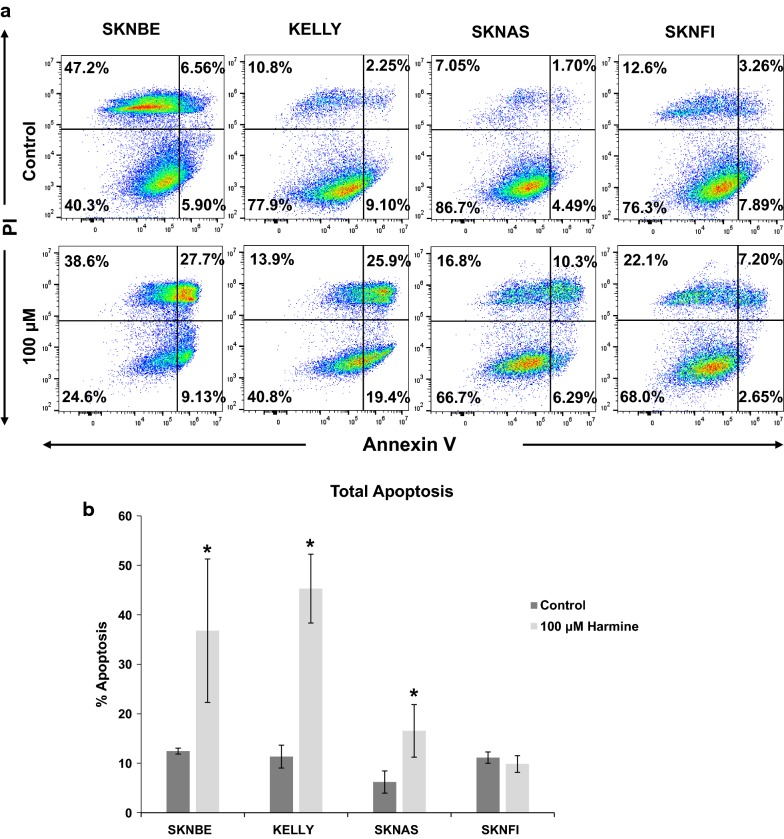
Harmine induces apoptosis, marked by increase in Annexin V-positive NB cells. **a** NB cell lines (SKNBE, KELLY, SKNAS, and SKNFI) were incubated with 100 μM harmine for 24 h, after which Annexin V presence was analyzed by flow cytometry using the Annexin V Apoptosis Detection Kit APC. The numbers in each quadrant represent the average number of events of three experiments (n = 3). **b** Bar graph representation showing an increase in the percentage of total apoptotic cells with harmine treatment. Data represent the average of three experiments ± S.D. (n = 3). Asterisk denotes a statistically significant increase in the percentage of apoptotic cells compared to control. The p-values are as follows: SKNBE P = 4.4 × 10^−2^; KELLY P = 1.3 × 10^−3^; and SKNAS P = 3.6 × 10^−2^. The p-value of SKNFI cells was not significant

To further confirm actual apoptosis in the harmine-treated NB cells, the amount of apoptotic cells in four NB cell lines was quantified using Annexin V staining and flow cytometry (Fig. 5a). The total amount of apoptotic cells in the SKNBE cells significantly increased from 12.5% ± 0.60 (control) to 36.8% ± 14.5 in cells that had been treated with harmine (100 μM) (Fig. 5b). Similarly, harmine significantly increased the total amount of apoptotic cells in KELLY cells from 11.3% ± 2.31 (control) to 45.3% ± 6.96 in the presence of harmine (100 μM). The change in apoptotic cells in SKNAS cells was less dramatic and increased from 6.20% ± 2.24 (control) to 16.6% ± 5.31 (100 μM) and there was no change in the percentage of apoptotic cells in SKNFI cells (Fig. 5b). The results further confirm our observation that harmine appears more toxic to *MYCN*-amplified NB cells (SKNBE, KELLY) than to NB cells with a normal *MYCN* gene copy number (SKNAS, SKNFI).

### Molecular docking model predicts interaction of DYRK2 with harmine

**Fig. 6:**
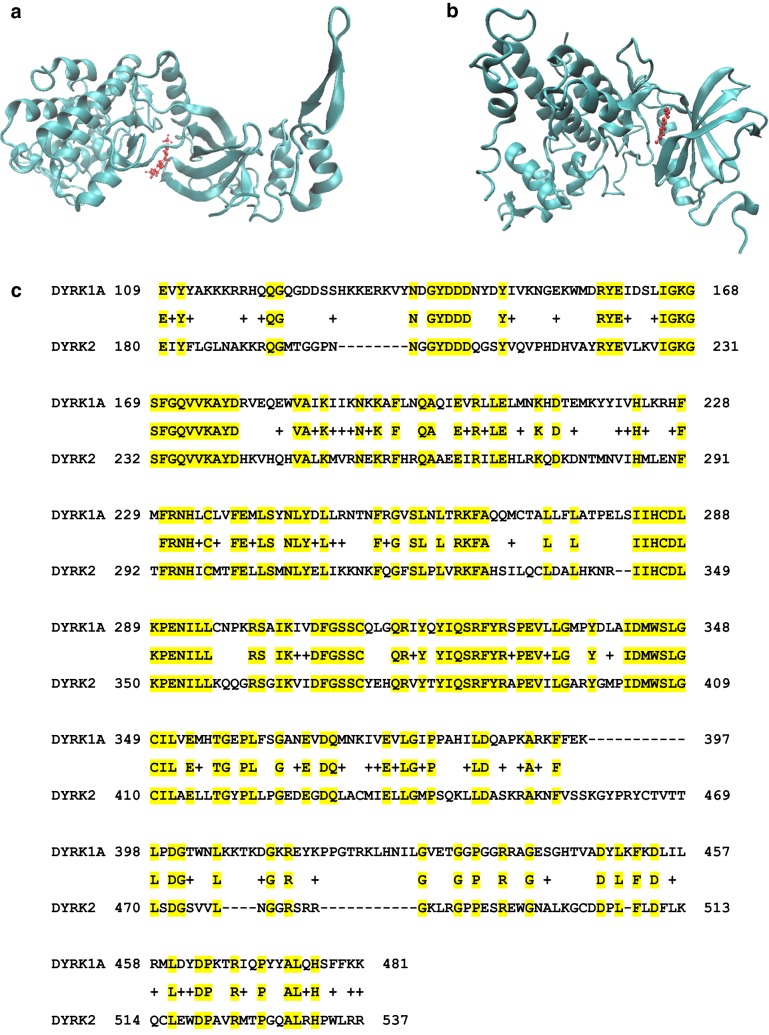
Molecular interaction of harmine with DYRK2. **a** The crystal structure of human DYRK1A chain A bound to harmine (PDB ID: 3ANR), as previously published [13]. Chain A is colored blue, and the structure of harmine is colored red. **b** Crystal structure of human DYRK2 chain A (PDB ID: 4AZF, ligand removed), as previously published [21] and docked with harmine. Chain A is shown in blue, and harmine is colored red. The figure was created using SwissDock to dock the structure of harmine (ZINC ID: 27646846), as detailed on the ZINC molecule database, to the published structure of chain A of the human DYRK2 protein. The resulting conformation had a ΔG value of − 6.89 kJ mol^−1^ and was deemed best fit by the parameters set by SwissDock. **c** BLAST sequence alignment of DYRK1A (Accession No. Q13627) and DYRK2 (Accession No. AAH06375) using the NCBI Blastp online tool [24]. The query cover was 51%, with an identity of 41% and an E-value of 4 × 10^−92^. Identical amino acids between the two sequences have been highlighted

Based on literature supporting the potential interaction between harmine and DYRK2, we performed a docking simulation using SwissDock [19, 20]. To accomplish this, we utilized the crystal structures of human DYRK1A in complex with harmine (PDB ID: 3ANR) (Fig. 6a) and human DYRK2 in complex with Leucettine (PDB:4AZF) [13, 21]. Chain A of human DYRK2 was isolated and was entered into the SwissDock portal with harmine as the ligand, after removing Leucettine. The structure of harmine was provided by the ZINC molecular database (ZINC ID: 27646846). The most favorable conformation of the DYRK2/harmine complex had a ΔG value of − 6.89 kJ mol^−1^ (Fig. 6a). The figure displays the highest affinity binding between human DYRK2 and harmine. The crystal structure of chain A of DYRK1A in complex with harmine is provided for comparison (Fig. 6b). The two proteins were also compared by performing a sequence alignment between human DYRK1A (Accession No. Q13627) and human DYRK2 (Accession No. AAH06375) using the NCBI Blastp online tool (Fig. 6c) [24]. The query identity was 41%, with an E-value of 4 × 10^−92^.

### *DYRK* family genes are expressed in NB tumors and *DYRK1B/2/3* predict poor patient outcome

**Fig. 7:**
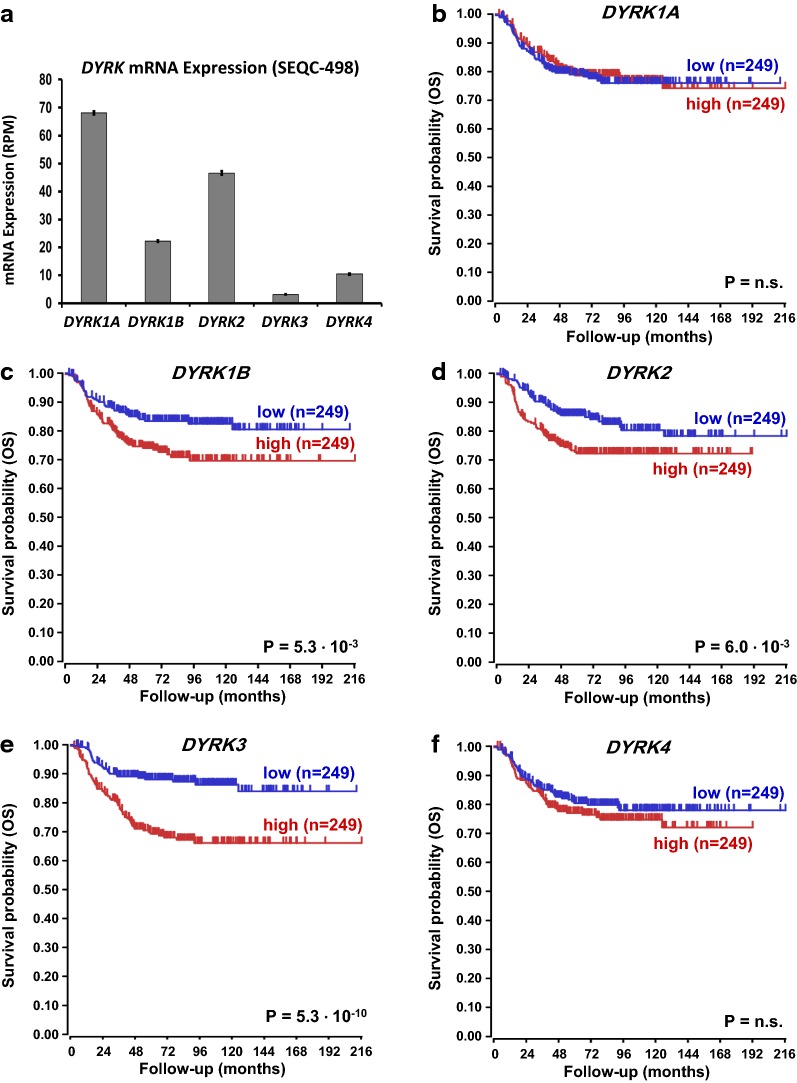
*DYRK* gene NB tumor mRNA expression and prognostic significance. The mRNA expression of the *DYRK* gene family was analyzed in SEQC-498, the largest genome-wide RNA sequencing dataset on human NB tumors in the public domain. **a** Bar plot of *DYRK* gene tumor mRNA expression in the SEQC-498 patient cohort. The Y-axis represents expression in RPM (reads per million), vertical bars represent mean expression ± standard deviation. All five *DYRK* genes show significant expression, with *DYRK1A* and *DYRK2* as the most highly expressed mRNAs. **b**–**f** Kaplan–Meier graphs representing the overall survival prognosis of NB patients in the SEQC-498 cohort based on grouping of the patients according to median *DYRK* gene tumor mRNA expression. **c**–**e** High-level tumor mRNA expression of *DYRK1B*, *DYRK2*, and *DYRK3* is significantly predictive of poor patient outcome. Significant correlations are also found when patients are grouped according to, e.g. average *DYRK* mRNA tumor expression, or when event-free survival is predicted (results not shown), suggesting that *DYRK* mRNA expression is a robust predictor for NB patient outcome

Our in vitro data in NB cell lines were supported by crystal structure-derived computational docking models and suggest that targeting DYRK family kinases through harmine treatment may be a potential new route of therapy for patients with NB. This notion prompted us to investigate DYRK family kinases in human NB tumors. To accomplish this, we analyzed the mRNA expression of the *DYRK* gene family in SEQC-498, the largest RNASeq dataset on human NB samples in the public domain. Analyses were performed using the R2 website (see Materials and Methods). We first determined whether *DYRK* genes are expressed in human NB tumors (Fig. 7a). All five *DYRK* genes show robust expression, especially *DYRK1A* and *DYRK2*.

**Fig. 8:**
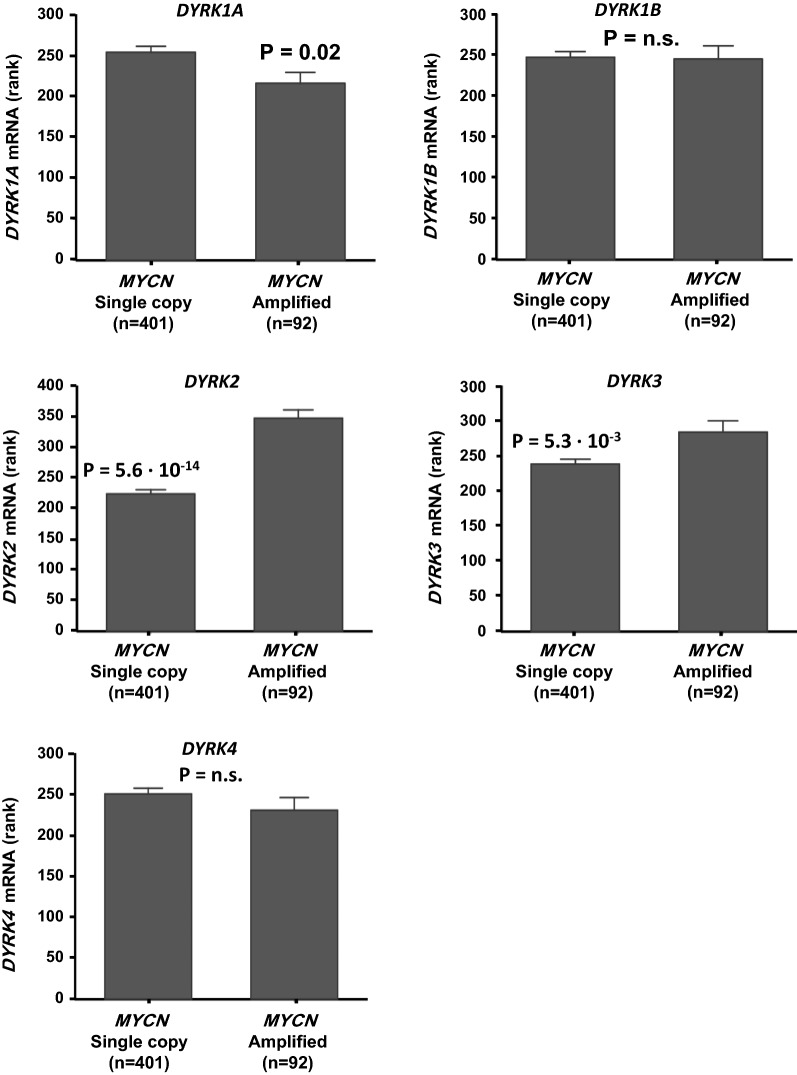
*DYRK* gene NB tumor expression correlated to *MYCN* gene amplification. The mRNA expression of the *DYRK* gene family was analyzed in SEQC-498 and correlated to *MYCN* amplification by separating the tumors into samples with normal *MYCN* gene copy number (n = 401) and with *MYCN* amplification (n = 92) and comparing the groups using the non-parametric Kruskal–Wallis test. The Y-axis represents *DYRK* gene mRNA expression (rank-based). The results show that *DYRK1A* mRNA expression is highest in tumors without *MYCN* amplification, but that both *DYRK2* and *DYRK3* are most highly expressed in tumors with *MYCN* amplification. *DYRK1B* and *DYRK4* expression are not correlated to *MYCN* gene copy number

We next determined whether high *DYRK* gene expression is beneficial to NB tumorigenesis and translates into poor patient outcome (Fig. 7b–f). Kaplan–Meier graphs representing the overall survival prognosis of NB patients in the SEQC-498 cohort based on grouping of the patients according to median *DYRK* gene tumor mRNA expression showed that high-level tumor mRNA expression of *DYRK1B*, *DYRK2*, and *DYRK3*, but not of *DYRK1A* or *DYRK4*, is significantly predictive of poor patient outcome (Fig. 7b–f). Although *DYRK1A* and *DYRK2* are the most highly expressed, only *DYRK2* is prognostic of survival outcome, suggesting that in NB the deleterious effect of harmine treatment acts mostly through *DYRK*2. Harmine toxicity tests and apoptosis analyses on NB cell lines suggest that *MYCN*-amplified cell lines are more sensitive to harmine than NB cell lines with two *MYCN* gene copies. We were interested whether this was reflected in the correlation between *DYRK* gene mRNA expression and *MYCN* tumor amplification in human NB tumors. We found that *DYRK2* and *DYRK3* mRNA expression in NB tumors was significantly higher in samples with *MYCN* gene amplification (Fig. 8). In contrast, *DYRK1B* and *DYRK4* tumor expression was not correlated to *MYCN* amplification status, and *DYRK1A* was actually lower in tumors with *MYCN* amplification. These results might indicate that the most aggressive, *MYCN*-amplified NB tumors that have the highest *DYRK2* and *DYRK3* expression could be more sensitive to harmine treatment, similar to the results we found in NB cell lines. These expression profiles along with the harmine-DYRK2 interaction model would suggest that the cytotoxic effect of harmine in NB is primarily through DYRK2 inhibition.

## Discussion

NB is a rare but lethal childhood tumor. Patients often present with advanced disease with survival chances below 50%, in spite of aggressive, multimodal therapy [9]. Almost all survivors of high-stage NB suffer from late therapy effects that severely impact quality of life. Current treatment might have reached a therapeutic plateau, clarifying an urgent need for more specific, more effective, novel therapies. The use of gene-targeting treatments is still very rare, and no current therapies target MYCN, the most important NB oncogene, that is almost invariantly over-expressed in advanced NB.

We investigated the use of harmine, a tricyclic β-carboline alkaloid with known, dose-dependent in vitro cytotoxicity for cell lines from different cancer types [9–11]. Harmine has been shown to inhibit MAO-A activity, increase BDNF levels, and inhibit human topoisomerase I [6, 27]. It is also a potent inhibitor of the DYRK family that interferes with neurite formation and DYRK1A plays a role in neurodegenerative disorders [14]. Although harmine displayed the highest in vitro potency against recombinant DYRK1A, it also inhibited DYRK1B and DYRK2, and, at much lower potency, DYRK4 [14]. Recently, harmine was found to synergize with the anthracycline doxorubicin in the breast cancer cell line MCF-7 [28] and harmine-mediated inhibition of DYRK1A destabilized epidermal growth factor receptor (EGFR) in aggressive glioblastomas and reduced EGFR-dependent glioblastoma growth [29]. Although harmine clearly binds to and inhibits DYRK proteins, it cannot be entirely ruled out that its anticancer effects at least in part were influenced by the inhibition of other potential harmine targets such as MAO-A or topoisomerase.

We observed strong cell death in harmine-treated NB cell lines that acting in part through the activation of caspase-3/7 and caspase-9. PARP cleavage and Annexin V staining analyses showed that apoptosis progressed into late stages. Further increasing the dose range of harmine resulted in similar effects (Additional file 1: Fig. S2). Excitingly, late apoptosis was significantly more prevalent in SKNBE and KELLY, two NB cell lines with *MYCN* amplification and concomitant MYCN oncoprotein expression that represent high-stage NB, than in two NB cell lines, SKNAS and SKNFI, with normal *MYCN* copy number and without MYCN over-expression. It has been previously shown that MYCN has the ability to promote apoptosis and specific targeted therapy combinations have been tested to exploit the apoptosis-primed state of *MYCN*-amplified NB cells. Moreover, caspases have been shown to contribute to cell cycle regulation independent of apoptosis, and caspase inhibitors prevented cell proliferation through induction of cell cycle arrest at mitotic phase [30, 31]. Our observations in vitro were further supported by mRNA expression data we generated in human NB tumors. We found that *DYRK2* and *DYRK3*, both harmine targets, are most highly expressed in NB patients with poor prognosis, and show *MYCN*-correlated expression. Although the expression of *DYRK1A* was also high in NB, it was not of prognostic value. These results strongly support our in vitro findings.

**Fig. 9:**
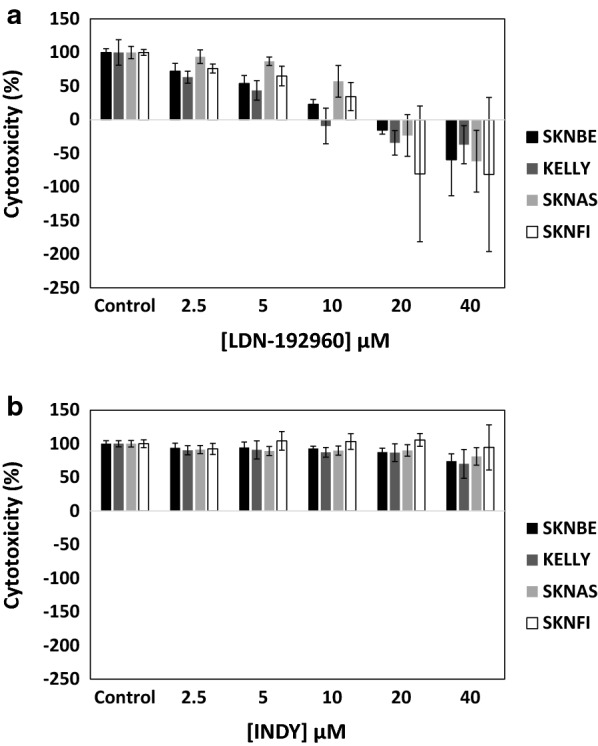
Cytotoxic effects of DYRK inhibitors after 24 h of treatment in NB cells using the SRB assay. **a** The DYRK2 inhibitor LDN-192960 strongly inhibited cell proliferation of four NB cell lines SKNBE, KELLY, SKNAS, and SKNFI in a dose-dependent manner. **b** In contrast, the DYRK1A/B inhibitor INDY only minimally inhibited cell proliferation under identical conditions. The time zero (T = 0) values for each cell line prior to treatment were subtracted from the 24 h readings. Data represent the average of three independent experiments, each performed in duplicate ± S.D. (n = 6)

To verify the importance of DYRK2 in NB, we treated four NB cell lines SKNBE, KELLY, SKNAS, and SKNFI with LDN-192960, a DYRK2/haspin inhibitor and INDY, a DYRK1A/B inhibitor [13, 32]. Strikingly, inhibition of DYRK2 with LDN-192960 induced significant cytotoxic effects whereas inhibition of DYRK1A/B with INDY had no effect under identical conditions (Fig. 9), thus underlining the importance of DYRK2 in NB.

## Conclusions

The childhood tumor NB is responsible for 16% of childhood cancer deaths. Therapy for high-stage disease that is often accompanied by tumor MYCN over-expression, is not sufficiently effective, and causes frequent severe late effects. The use of the natural product harmine that targets DYRK family proteins to treat NB cell lines in vitro showed extensive cytotoxicity, especially in cell lines with *MYCN* gene amplification and over-expression. These experimental data combined with our in silico modeling and epidemiologic data strongly suggest a role for DYRK2 in NB tumorigenesis and the potential for harmine as an effective treatment option for *MYCN*-amplified NB.

## Additional file

**Additional file 1: Table S1.** IC50 = micromolar (μM) concentration at which harmine inhibits 50% of viable cells. +/− standard deviation (n = 3). N/D = not done (50% inhibition was not reached). **Fig. S1.** Micrographs depicting the effects of 100 μM harmine on four different human neuroblastoma cell lines. Pictures were taken at 24, 48, and 72 h after exposure to the harmine. The 72 h micrographs are included as a part of Fig. 2. **Fig. S2.** PARP cleavage was detected after 24 h of harmine treatment at increasing concentrations (0–200 μM) using Western blot. Higher concentrations of harmine are required to detect the cleavage of PARP in the SKNAS and SKNFI cell lines after a 24 h treatment. Data are representative of three independent experiments (n = 3).

## Abbreviations

DYRK: dual-specificity tyrosine phosphorylation-regulated kinase
NB: neuroblastoma
