# Supplementary material for: Harmine, a dual-specificity tyrosine phosphorylation-regulated kinase (DYRK) inhibitor induces caspase-mediated apoptosis in neuroblastoma
Source: Cancer Cell Int. 2018 Jun 7;18:82. doi: 10.1186/s12935-018-0574-3 (PMC5992763; doi:10.1186/s12935-018-0574-3)
Supplement: Supplementary file 1 — Additional file 1: Table S1. IC50 = micromolar (μM) concentration at which harmine inhibits 50% of viable cells. +/− standard deviation (n = 3). N/D = not done (50% inhibition was not reached). Fig. S1. Micrographs depicting the effects of 100 μM harmine on four different human neuroblastoma cell lines. Pictures were taken at 24, 48, and 72 h after exposure to the harmine. The 72 h micrographs are included as a part of Fig. 2. Fig. S2. PARP cleavage was detected after 24 h of harmine treatment at increasing concentrations (0–200 μM) using Western blot. Higher concentrations of harmine are required to detect the cleavage of PARP in the SKNAS and SKNFI cell lines after a 24 h treatment. Data are representative of three independent experiments (n = 3). [file 12935_2018_574_MOESM1_ESM.pdf]

Supplementary Information

	24 hours	48 hours	72 hours
**SKNBE**	558.7 ± 1.10	224.8 ± 0.16	169.6 ± 0.10
**KELLY**	3395 ± 7.90	238.7 ± 0.13	170.8 ± 0.10
**SKNAS**	635.6 ± 0.70	N/D	N/D
**SKNFI**	2696 ± 4.40	N/D	791.7 ± 0.77

Table S1. IC_{50} = micromolar (μM) concentration at which harmine inhibits 50% of viable cells. +/- standard deviation (n=3). N/D = not done (50% inhibition was not reached).

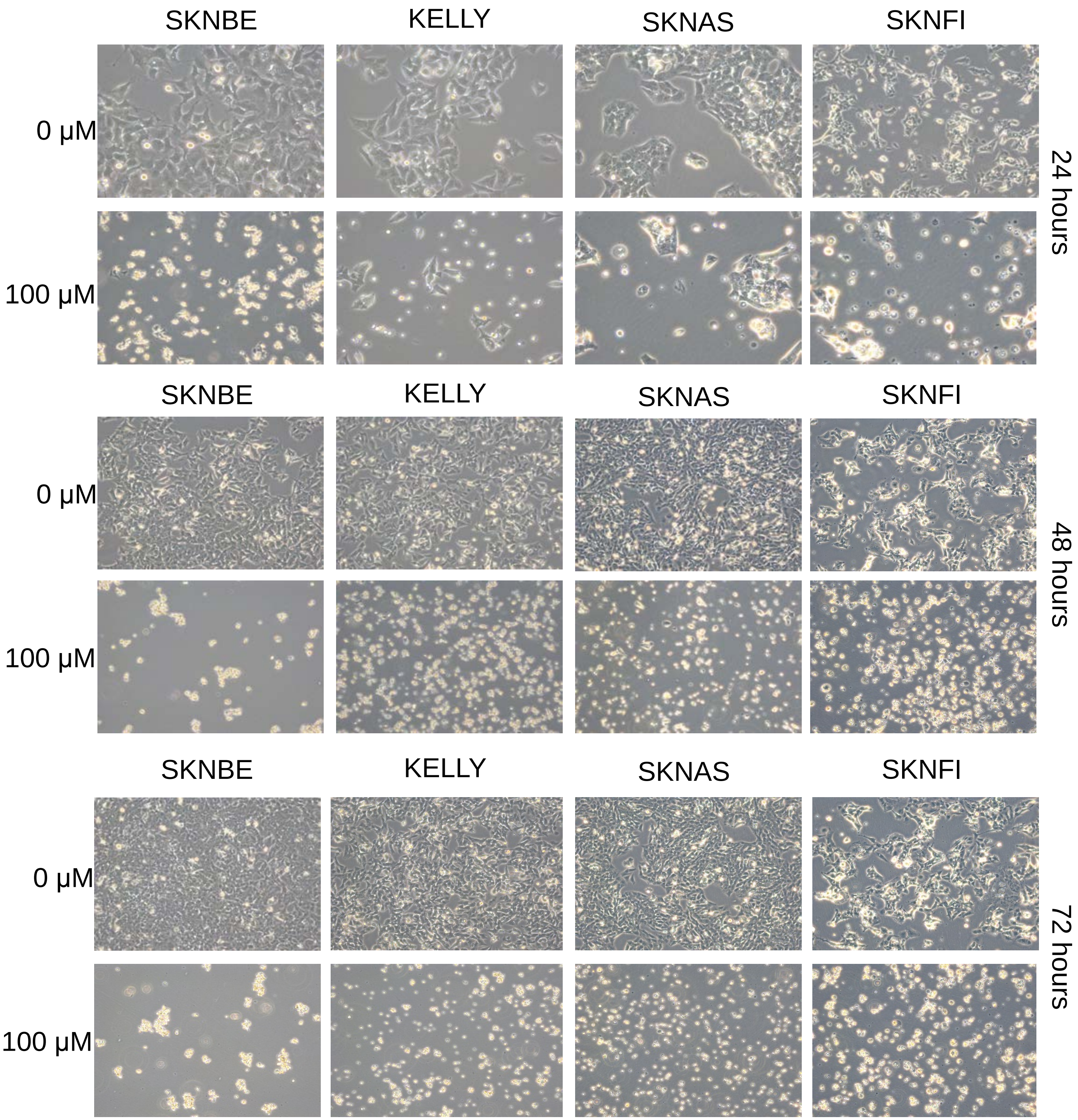

Fig. S1. Micrographs depicting the effects of 100 μM harmine on four different human neuroblastoma cell lines. Pictures were taken at 24, 48, and 72 hours after exposure to the harmine. The 72 hours micrographs are included as a part of Figure 2.

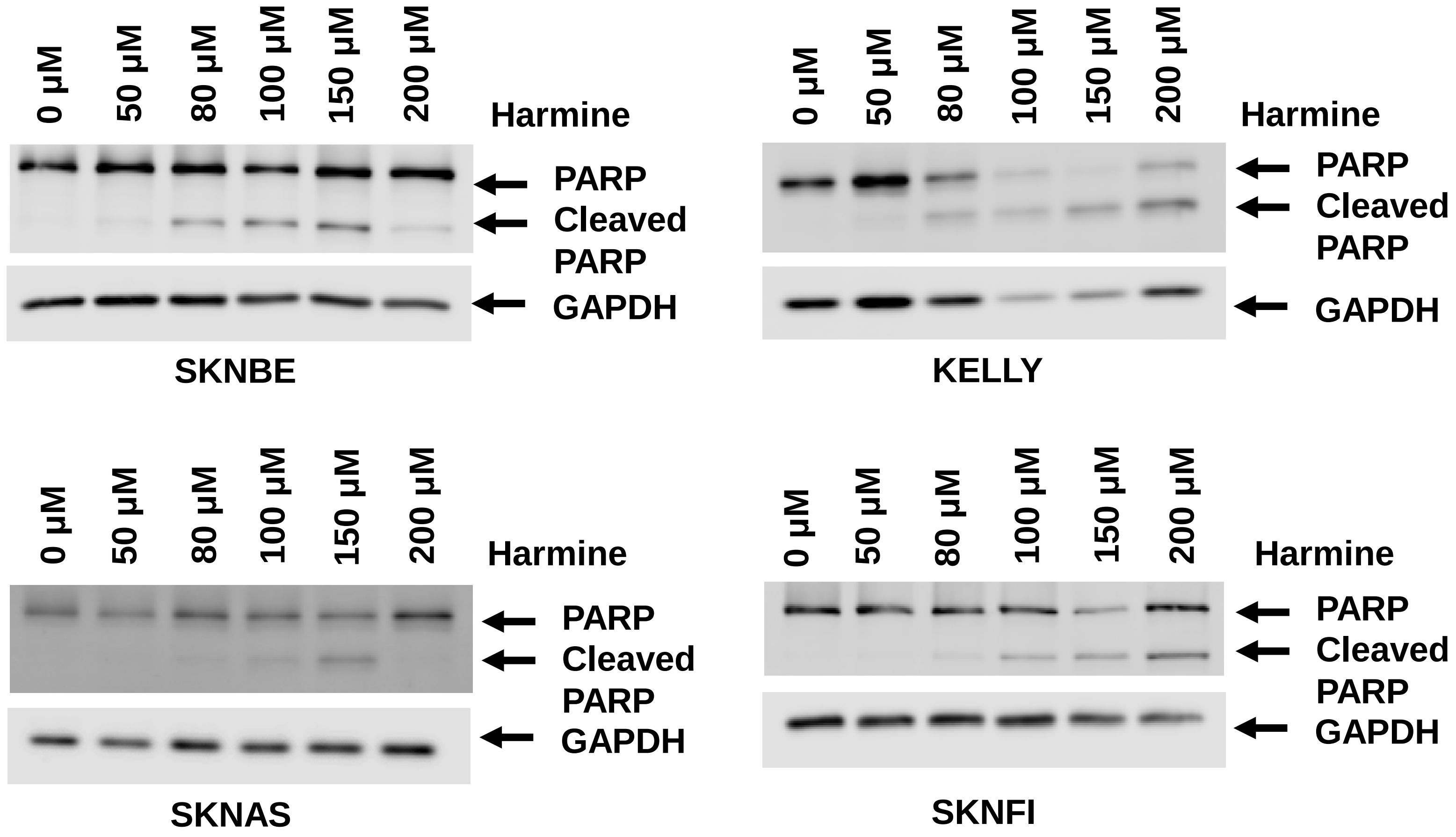

Fig. S2. PARP cleavage was detected after 24 hours of harmine treatment at increasing concentrations (0-200 µM) using Western blot. Higher concentrations of harmine are required to detect the cleavage of PARP in the SKNAS and SKNFI cell lines after a 24 hour treatment. Data are representative of three independent experiments (n=3).
